# Supplementary material for: Identification of the governing equation of stimulus-response data for run-and-tumble dynamics
Source: PLoS Comput Biol. 2025 Aug 5;21(8):e1013287. doi: 10.1371/journal.pcbi.1013287 (PMC12338844; doi:10.1371/journal.pcbi.1013287)
Supplement: S1 Text — (PDF) [file pcbi.1013287.s001.pdf]

Supporting Information:

Identification of the governing equation of stimulus-response data for run-and-tumble dynamics

Shicong Lei, Yu'an Li, Zheng Ma, Hepeng Zhang, Min Tang

July 25, 2025

Appendix A. Details of Model I, II, and III

Model I. Model I is a single-internal-variable model for *E. coli* signal processing that is developed in [1]. The dynamics of m incorporate the methylation and demethylation reactions such that

$$\frac{dm}{dt} = k_R R(1 - A(m,s)) - k_{Bp} B_p A(m,s), \tag{A.1}$$

where R and B_p denote the concentration of two proteins CheR and CheBp, respectively. Here k_R and k_{Bp} are respectively the methylation and demethylation rates, $A(m,s)$ is the receptor activity that is determined by:

$$\begin{aligned} A(m,s) &= \frac{1}{1+\exp\left(N_r F_A(m,s)\right)}, \\ F_A(m,s) &= \alpha\left(m_0 - m\right) + \ln\left(\frac{1+s/K_I}{1+s/K_A}\right). \end{aligned} \tag{A.2}$$

In Eq (A.2), m_0 is a reference methylation level; α measures how the free energy of the receptor complex depends on m; K_I and K_A are respectively the signal dissociation constants for inactive and active receptors; and N_r is the average number of nearest neighbors of the receptor functioning units. The protein concentrations R and B_p are implicitly given by the following system of quasi-steady-state equations:

$$\begin{aligned} & k_A\left(T_t A(m,s) - T_p\right) - k_Y Y T_p - k_B B T_p = 0, \\ & k_Y Y T_p - \mu_Y Y_p - k_Z Z Y_p = 0, \\ & k_B B T_p - \mu_B B_p = 0, \\ & Y = \frac{Y_t - \left(1+K_Z Z\right) Y_p}{1+K_Y T_p}, \quad Z = \frac{Z_t}{1+K_Z Y_p}, \\ & B = \frac{B_t - \left(1+K_{Bp} T_t A(m,s)\right) B_p}{1+K_B T_p}, \quad R = \frac{R_t}{1+K_R T_t(1-A(m,s))}. \end{aligned} \tag{A.3}$$

Here, Y_p and T_p are the concentrations of CheYp and CheAp-associated receptors; the constants T_t, Y_t, B_t, Z_t, and R_t are the total concentrations of the corresponding proteins; ks, K s, and μs are reaction rates of the corresponding proteins. The parameter values for Model I are the same as in [1,2] and are summarized in Table A.

Model II. In [2,4], the dynamics of the CheY-P concentration Y_p and receptor methylation level m are modeled by:

$$\begin{aligned} \frac{dm}{dt} &= k_R\left(1 - A(m,s)/a_0\right), \\ \frac{dY_p}{dt} &= k_a A(m,s) - Y_p/\tau_Z. \end{aligned} \tag{A.4}$$

Table A. Parameters of Model I. Parameter used for $A(m, s)$ are taken from [2]. Z_t is from [3]. Other parameters are consistent with [1].

Parameter	Description	Values
m_0	Reference methylation level in the absence of the input signal	1
α	Measurement of how the free energy of the receptor complex depends on m	1.7
K_I	Signal dissociation constants for inactive receptors	18 µM
K_A	Signal dissociation constants for active receptors	3 mM
N_r	Average number of nearest neighbors of the receptor functioning units	6
k_R	Methylation rate mediated by CheR	3.82×10^{-2} s^{-1}
k_{B_p}	Demethylation rate mediated by CheBp	3.25 s^{-1}
k_A	Phosphorylation rate of T_p mediated by CheA	100 s^{-1}
k_Y	Dephosphorylation rate of T_p mediated by CheY	130 µM^{-1}s^{-1}
k_B	Dephosphorylation rate of T_p mediated by CheB	7.5 µM^{-1}s^{-1}
k_Z	Dephosphorylation rate of Y_p mediated by CheZ	8.45 µM^{-1}s^{-1}
μ_Y	Degradation rate of Y_p	0.1 s^{-1}
μ_B	Degradation rate of B_p	1 s^{-1}
K_B	Association constant for CheB phosphorylation	0.25 µM^{-1}
K_{B_p}	Association constant for receptor demethylation	6.5 µM^{-1}
K_R	Association constant for receptor methylation	0.15 µM^{-1}
K_Y	Association constant for CheY phosphorylation	0.65 µM^{-1}
K_Z	Association constant for CheYp dephosphorylation	1 µM^{-1}
B_t	Total concentration of CheB	2 µM
R_t	Total concentration of CheR	0.3 µM
T_t	Total concentration of CheT	5/3 µM
Y_t	Total concentration of CheY	18 µM
Z_t	Total concentration of CheZ	1.23 µM

Here k_a is the phospho-transfer rate from active CheA to CheY, and τ_Z is the dephosphorylation time. The definitions and forms of $A(m, s)$ in Model II are identical to Eq (A.2) in Model I (parameters used in $A(m, s)$ are shown in Table A). The parameter values for the model are summarized in Table B.

Table B. Parameters of Model II. k_R [1], a_0 [2], and τ_Z [4]. k_a is adapted for the simulation based on the response value.

Parameter	Description	Values
k_R	Methylation rate mediated by CheR	3.82×10^{-2} s^{-1}
a_0	Coefficient in Eq (A.4)	0.5
k_a	Phospho-transfer rate from active CheA to CheY	10 s^{-1}
τ_Z	Dephosphorylation time	0.5 s

The tumbling fraction f in Models I and II is determined by the concentration of CheYp. Each *E. coli* cell has several flagella and each flagellar motor can rotate either clockwise (CW) or counter-clockwise (CCW). The switching rates λ_f

(from CCW to CW) and μ_f (from CW to CCW) depend on the intracellular CheYp level Y_p such that [3]:

$$\begin{aligned}\lambda_f &= a_1 \exp\left(b_1 Y_p\right)\\ \mu_f &= a_2 \exp\left(-\left(b_2 - Y_p\right)^4/c\right)\end{aligned} \tag{A.5}$$

where a_1, b_1, a_2, b_2 and c are constants. Under the assumptions that each cell has n_f flagella that rotate independently and the cell runs forward If at least w flagella rotate CCW simultaneously, The probability for the cell to be in the run state is given by

$$P_{\text{run}} = \sum_{i=w}^{n_f} P^i_{CCW} = \sum_{i=w}^{n_f} \left(\begin{array}{c} n_f \\ i \end{array} \right) \left(\frac{\mu_f}{\lambda_f + \mu_f}\right)^i \left(\frac{\lambda_f}{\lambda_f + \mu_f}\right)^{n_f - i}. \tag{A.6}$$

Consequently, the tumbling fraction is given by

$$f = P_{\text{tumble}} = 1 - P_{\text{run}}. \tag{A.7}$$

Details of all parameters are the same as in [3] and given in Table C.

Table C. Parameters of the response function in Eq (A.5-A.7). All parameters are consistent with [3]

Parameter	Description	Values
a_1	Coefficient in Eq (A.5)	0.0174001 s^{-1}
b_1		1.32887 μM^{-1}
a_2		12.0809 s^{-1}
b_2		-5.83762 µM
c		2892.12
n_f	Total flagella number	8
w	Minimum number of CCW flagella needed to run	6

Model III. In [5], the dynamics of FliM proteins in the c-ring FliM and receptor methylation level m can be described by:

$$\begin{aligned}\frac{dm}{dt} &= V_R(1 - A(m,s)) - V_B A(m,s),\\ \frac{d}{dt}\text{FliM} &= V_{\text{on}}\ (\text{FliM}_{\text{MAX}} - \text{FliM}) - V_{\text{off}}\, A(m,s)\text{FliM}.\end{aligned} \tag{A.8}$$

The definitions and forms of $A(m,s)$ in Model III are identical to Eq (A.2) in Model I. FliM_{MAX} is the maximum number of FliM units that can be in the c-ring. V_{on} and V_{off} are association and dissociation rates of FliM to the c-ring. The dependence of the motor tumbling bias f is given by the following modified Hill function:

$$f = \frac{(1 + A/Q)^{k_1 * (\text{FliM} - k_2)}}{(1 + A/Q)^{k_1 * (\text{FliM} - k_2)} + P\left(1 + A/\left(QC\right)\right)^{k_1 * (\text{FliM} - k_2)}}, \tag{A.9}$$

where P is the ratio of the probability that the motor is in the CCW state to the probability that it is in the CW state in the absence of CheY-P; C is the ratio of CheY-P dissociation constants for the CCW and CW states; k_1 and k_2 are constants. The parameters of Model III are shown in Table D.

Appendix B. *E. gracilis* culture and experiment setup

***E. gracilis* culture.** *E. gracilis*, from Caralina Biological Supply Company, is cultured in modified Cramer-Myers medium, which is placed in an orbital shaker operating at 70r/min inside an incubator (Yiheng MGC-100BP) set to 20°C.

Table D. Parameters of Model III. k_1 **and** k_2 **are adjusted to amplify overshoot in the step response. Other parameters are taken from [5].**

Parameter	Description	Values
V_R	Methylation rate mediated by CheR	0.01 s^{-1}
V_B	Demethylation rate mediated by CheB	0.02 s^{-1}
V_{off}	Dissociation rates of FliM to the c-ring	1.94 $\times 10^{-2}$ s^{-1}
V_{on}	Association rates of FliM to the c-ring	0.02 s^{-1}
FliM_{MAX}	Maximum number of FliM units in the c-ring	45
P	Ratio of running probability to tumbling probability in the absence of CheY-P	10^7
C	Ratio of CheY-P dissociation constants for the CCW and CW states	4.1
Q	Coefficient in Eq (A.9)	0.35
k_1		8
k_2		30

A 24-hour light cycle is applied, consisting of 12 hours of cool light with an intensity of 7W/m^2 followed by 12 hours of darkness. For all experiments, cells aged between 14-20 days are used, ensuring optimal motility and photo-responsiveness. Prior to experiments, cells are acclimated overnight in the incubator. During experiments, cell culture is placed in a custom-built sample chamber (as shown in Fig A), which is made by two cover slides sealed with double-sided tapes (150µm).

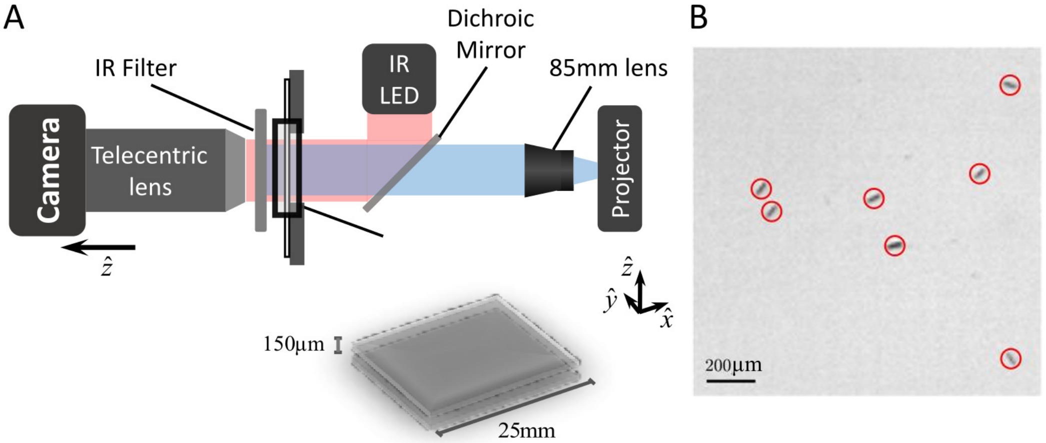

Fig A. ***E. gracilis*** **experiment setup and data measurements.** (A) An overview of the experimental setup. A projector generates a blue light pattern to manipulate the algae's motion, while infrared light uniformly illuminates the entire chamber for video recording. The cell movements are captured using a 4-megapixel camera paired with a telecentric lens, providing an extensive depth of field of up to 1mm, exceeding the boundaries of the sample chamber. (B) Raw experimental image. Red circles highlight seven cells.

Experiment setup. Blue light patterns (wavelength: 470nm) are projected into the sample chamber to control cell motion, while infrared illumination (wavelength: 850nm) provides uniform lighting for imaging without affecting the cells' phototactic response [6–8]. The patterns are generated by a Texas Instruments projector (DLP4710G2) and focused using an 85mm lens, with intensity modulated via LED current adjustment or blue-channel tuning of the input video. A digital optical power meter (DHC GCI-080102) measures the precise intensity at the sample plane. Cell motion is recorded at 10 frames per second using a Basler area scan camera (acA2040-90um, 4 MP) fitted with a telecentric lens (ESCM05-110X20,

0.5× magnification). To prevent external interference, all experiments are conducted in a dark room maintained at 25°C.

Data measurements. Cell orientation and position are extracted using an image analysis pipeline based on intensity thresholding. Cell trajectories are then reconstructed using a particle-tracking algorithm employing a nearest-neighbor linking strategy across successive frames. Tumbling is defined as a decrease in translational velocity or a concurrent increase in angular velocity. To identify tumbling events, we adapt the algorithm proposed by Masson et al. [9], which relies on analyzing temporal changes in cell velocity and angular speed. After identifying the tumbling events in each frame, the tumbling fraction is calculated as the ratio of tumbling particles to the total number of detected particles in that frame. To obtain a time-resolved measure, the tumbling fraction is averaged over 10 frames (corresponding to one second), resulting in a final dataset with one data point per second.

Appendix C. Theoretical degeneration condition

We perform perturbation analysis to establish the conditions under which the system can be simplified—i.e., one can use a quasi-equilibrium approximation for the fast variable.

Assume that the dual internal variable reactions can be modeled by:

$$\frac{dm}{dt} = \frac{1}{\tau_m} H_1(m, n, s), \tag{C.1}$$

$$\frac{dn}{dt} = \frac{1}{\tau_n} H_2(m, n, s), \tag{C.2}$$

where τ_m and τ_n are respectively the reaction rates of m and n. When $\tau_n \ll \tau_m$, n is the fast variable and we consider n to be perturbed slightly from its quasi-equilibrium value n_{ss} that satisfies $H_2(m, n_{\mathrm{ss}}, s) = 0$. That is

$$n(t) = n_{\mathrm{ss}}(m(t), s(t)) + \delta n(t), \tag{C.3}$$

where $\delta n(t)$ represents the deviation from the quasi-equilibrium state. Taking the time derivative on both sides of (C.3) yields:

$$\frac{dn}{dt} = \frac{dn_{\mathrm{ss}}}{dt} + \frac{d\delta n}{dt}. \tag{C.4}$$

Since n_{ss} is a function of m and s, the total derivative of n_{ss} can be given by:

$$\frac{dn_{\mathrm{ss}}}{dt} = \frac{\partial n_{\mathrm{ss}}}{\partial m}\frac{dm}{dt} + \frac{\partial n_{\mathrm{ss}}}{\partial s}\frac{ds}{dt} = \frac{1}{\tau_m}\frac{\partial n_{\mathrm{ss}}}{\partial m} H_1(m, n, s) + \frac{\partial n_{\mathrm{ss}}}{\partial s}\frac{ds}{dt}.$$

Linearizing $H_2(m, n, s)$ about n_{ss} gives:

$$\begin{aligned} H_2(m, n, s) =& H_2(m, n_{\mathrm{ss}} + \delta n, s) \\ \approx& H_2(m, n_{\mathrm{ss}}, s) + \left.\frac{\partial H_2}{\partial n}\right|_{n_{\mathrm{ss}}} \delta n = \left.\frac{\partial H_2}{\partial n}\right|_{n_{\mathrm{ss}}} \delta n. \end{aligned} \tag{C.5}$$

Substituting (C.5) into the fast dynamics equation (C.2) and using (C.4), one has:

$$\frac{dn}{dt} = \frac{1}{\tau_n}\left.\frac{\partial H_2}{\partial n}\right|_{n_{\mathrm{ss}}} \delta n = \frac{dn_{\mathrm{ss}}}{dt} + \frac{d\delta n}{dt} = \frac{1}{\tau_m}\frac{\partial n_{\mathrm{ss}}}{\partial m} H_1(m, n, s) + \frac{\partial n_{\mathrm{ss}}}{\partial s}\frac{ds}{dt} + \frac{d\delta n}{dt}. \tag{C.6}$$

Since $\tau_n \ll \tau_m$ and δn varies slowly compared to the fast dynamics, the terms $\frac{1}{\tau_m}\frac{\partial n_{\mathrm{ss}}}{\partial m} H_1(m, n, s)$ and $\frac{d\delta n}{dt}$ can be neglected. Then from (C.6), the quasi-equilibrium state of δn is

$$\delta n \approx \frac{\frac{\partial n_{\mathrm{ss}}}{\partial s}\frac{ds}{dt}}{\frac{1}{\tau_n}\left.\frac{\partial H_2}{\partial n}\right|_{n_{\mathrm{ss}}}}. \tag{C.7}$$

We assume that the quasi-equilibrium approximation remains valid when $|\delta n|_{\max} < \Delta_n$, which indicates that:

$$\left| \frac{\tau_n \frac{\partial n_{\mathrm{ss}}}{\partial s} \frac{ds}{dt}}{\frac{\partial H_2}{\partial n}\Big|_{n_{\mathrm{ss}}}} \right| < \Delta_n$$

This leads to a restriction on the variation of external signals:

$$\left| \frac{ds}{dt} \right|_{\max} < \left| \frac{\tau_n \frac{\partial H_2}{\partial n}\Big|_{n_{\mathrm{ss}}} \Delta_n}{\frac{\partial n_{\mathrm{ss}}}{\partial s}} \right|_{\max} := S_g \tag{C.8}$$

For (A.4) in Model II, which incorporates a slowly varying methylation level (m) and a rapidly changing CheY-P concentration (n), the sensitivity metric S_g can be derived by setting $\Delta_n = 0.1$, $s \in [0, 2]$ µM and corresponding $m \in [1, 1.07]$ near the reference methylation level $m0$, reducing the above expression to:

$$S_g = \left| \frac{\frac{\partial H_2}{\partial n}\Big|_{n_{\mathrm{ss}}} \Delta_n}{\frac{\partial n_{\mathrm{ss}}}{\partial s}} \right|_{\max} = \frac{\Delta_n}{\tau_Z} \cdot \max_{m \in [1,1.07], s \in [0,2]} \left| \frac{(A(m,s))^{-2}(1 + s/K_I)}{N_r k_a \tau_Z \exp(N_r F_A(m,s)) \left(1/K_I - \frac{1+s/K_I}{K_A+s}\right)} \right| \approx 0.59 \text{ µM/s},$$

where $\tau_Z = 0.5$ s represents the characteristic timescale of Y_p and details of all other parameters can be found in Table B. This analytical result aligns with the numerically observed gradient threshold.

Appendix D. Settings for external stimuli and algorithm datasets

Settings for "Performance on the *E. coli* tumbling fraction". Here, we use stimulus-response data obtained by simulating Model I-III and then adding some noise. The stimuli $s(t)$ in the training, validation, and testing sets are of the form PWC, LCC, or ELCC, whose particular parameter values are chosen randomly from given intervals. The given intervals determine the magnitudes and variation rates of $s(t)$. The interval $[a_{\min}, a_{\max}]$ is determined to ensure that the magnitude of $s(t)$ remains within a specified range $[s_{\min}, s_{\max}]$, which remain the same for different data sets. The interval $[b_{\min}, b_{\max}]$ is determined by the variation rates.

For Models I and II, the adaptation time of the tumbling fraction is 10 to 30 seconds after a sudden change in stimulus (see Fig F(A-B)). For Model III, the focus is on the "overshoot" phenomenon during the adaptation phase (see Fig F(C)), which typically occurs 25 to 70 seconds after a sudden change in stimulus. Correspondingly, the details for each model's stimulus settings are as follows:

- Model I: All stimuli are within the range defined by $s_{\min} = 0$ and $s_{\max} = 0.5$. For both LCC and ELCC stimuli, the parameters are set as $a_{\min} = 0.15$, $a_{\max} = 0.25$, $b_{\min} = 10$, and $b_{\max} = 30$.
- Model II: All stimuli are within the range defined by $s_{\min} = 0$ and $s_{\max} = 2$. For LCC stimuli, the parameters are set as $a_{\min} = 0.6$, $a_{\max} = 1$, $b_{\min} = 10$, and $b_{\max} = 30$. For ELCC stimuli, the parameters are set as $a_{\min} = 0.15$, $a_{\max} = 0.25$, $b_{\min} = 10$, and $b_{\max} = 30$.
- Model III: All stimuli are within the range defined by $s_{\min} = 0$ and $s_{\max} = 2$. For LCC stimuli, the parameters are set as $a_{\min} = 0.6$, $a_{\max} = 1$, $b_{\min} = 25$, and $b_{\max} = 70$. For ELCC stimuli, the parameters are set as $a_{\min} = 0.15$, $a_{\max} = 0.25$, $b_{\min} = 25$, and $b_{\max} = 70$.

In order to get the performance on *E. coli* models in Fig 2, the details for each model's training, validation, and test sets are as follows:

- The training set includes PWC and LCC stimuli along with their smoothed response data. A total of $N^{\text{train}} = 13,900$ stimulus-response groups are used, with LCC data groups accounting for 58%.
- The validation set consists of new LCC stimuli and their smoothed response data, with $N^{\text{vali}} = 2,002$.
- The test set includes ELCC stimuli and their smoothed response data, with $N^{\text{test}} = 4,004$.

Settings for "Performance on the measured *E. gracilis* tumbling fraction". Due to the availability of the experimental data, to get Fig 3 and Table 1, the details for each experiment's stimulus settings are as follows:

- Slow: All stimuli are within the range defined by $s_{\min} = 0$ and $s_{\max} = 0.5$. For LCC stimuli, the parameters are set as $a_{\min} = 0.15$, $a_{\max} = 0.25$, $b_{\min} = b_{\max} = 300$.
- Fast: All stimuli are within the range defined by $s_{\min} = 0$ and $s_{\max} = 0.5$. For LCC stimuli, the parameters are set as $a_{\min} = 0.15$, $a_{\max} = 0.32$, $b_{\min} = 90$, and $b_{\max} = 250$.

The details for each experiment's training, validation, and test sets are as follows:

- Slow: For the training set, $N^{\text{train}} = 10,234$ stimulus-response groups are used, with 76% PWC and 24% LCC stimulus-response groups. For the validation set, $N^{\text{vali}} = 1,961$ stimulus-response groups are used, with 100% PWC stimulus-response groups. For the test set, $N^{\text{test}} = 2,370$ stimulus-response groups are used, with 50% PWC and 50% LCC stimulus-response groups.
- Fast: For the training set, $N^{\text{train}} = 8,248$ stimulus-response groups are used, with 42% PWCL and 58% LCC stimulus-response groups. For the validation set, $N^{\text{vali}} = 1,579$ stimulus-response groups are used, with 24% PWCL and 76% LCC stimulus-response groups. For the test set, $N^{\text{test}} = 1,967$ stimulus-response groups are used, with 39% PWCL and 61% LCC stimulus-response groups.

Settings for "Relation between SIVM and DIVM and criteria for their selection". We first regard stimuli used in Fig 2(B) as "baseline stimuli". To get Fig 4, $s(t)$ is progressively compressed by setting $T_1 = \{100, 120, 140, 160, 180\}$ respectively, and stimulus-response data sets are obtained by simulating Model II. The details for the training, validation, and test sets are consistent with those used in Fig 2(B).

Settings for "Design principles for stimuli". To get Table 2, $N^{\text{data}} = 20,020$ stimulus-response groups are used, with 70% allocated to the training set and the rest for testing. The details for each model's stimulus settings are as follows:

- Model I: All stimuli are within the range defined by $s_{\min} = 0$ and $s_{\max} = 0.5$. For both LCC stimuli, the parameters are set as $a_{\min} = 0.15$, $a_{\max} = 0.25$, $b_{\min} = 10$, and $b_{\max} = 30$.
- Model II: All stimuli are within the range defined by $s_{\min} = 0$ and $s_{\max} = 2$. For LCC stimuli, the parameters are set as $a_{\min} = 0.6$, $a_{\max} = 1$, $b_{\min} = 10$, and $b_{\max} = 30$.
- Model III: All stimuli are within the range defined by $s_{\min} = 0$ and $s_{\max} = 2$. For LCC stimuli, the parameters are set as $a_{\min} = 0.6$, $a_{\max} = 1$, $b_{\min} = 25$, and $b_{\max} = 70$.

For the three types of signals- PWC, PWCL, and LCC -the training set includes one, two, or all three of them. The proportions of each signal in the training sets and test sets are specified as follows:

- For the training set containing PWC and PWCL stimulus-response groups, each type of stimulus-response group accounts for 50%.
- For the training set containing LCC and either PWC or PWCL stimulus-response groups, LCC constitutes 58%, consistent with the settings of the *E. coli* numerical experiments.
- For the training set containing LCC, PWC, and PWCL stimulus-response groups, the proportions are 42%, 29%, and 29%, respectively.
- For the test set, three types of stimulus-response groups are evenly distributed.

Appendix E. Robustness tests

In this section, we investigate our algorithms' robustness to complex stimuli with stochastic fluctuations. Specifically, the noisy stimuli are given by

$$s_i = s_i^{\text{origin}} + \epsilon_i,\ \epsilon_i \sim \mathcal{N}\left(0, \sigma_\epsilon^2\right),\ i = 1, 2, \cdots, N^{\text{data}},$$

where s^{origin} represent noiseless PWL, LCC, and ELCC signals and σ_ϵ can be obtained through

$$\sigma_\epsilon = \sqrt{P_{\text{noise}}} = \sqrt{\frac{P_{\text{signal}}}{10^{\text{SNR}/10}}}.$$

with SNR following the uniform distribution $U(20, 22)$. We generate tumbling responses f_i by simulating Model I-III using a forward Euler discretization based on these noisy stimuli (as shown in Fig B). Clearly, response sequences derived from noisy stimuli exhibit fluctuations, the magnitude of which is determined by each model's dynamical equations. Detailed settings for Model I-III's training, validation, and test sets are the same as settings for "Performance on the *E. coli* tumbling fraction" shown in Appendix D.

Noisy data cannot be accurately differentiated and also requires prior smoothing. The cubic smoothing spline method (see subsection 1 in "Results") is applied to smooth the data sequences $s(t)$ and $f(t)$, from which the derivatives f_i', s_i', f_i'', and s_i'' are approximated. It should be noted that, during both training and testing, we utilize noisy stimulus-response data (f_i, s_i), which contrasts with subsection 1 in "Results".

For testing, we compare the predicted dynamics $\hat{f}_i$ inferred by both algorithms with f_i. The prediction accuracy is quantified using the relative mean squared error:

$$E_{\text{test}} = \frac{1}{N^{\text{test}}} \sum_{i=1}^{N^{\text{test}}} \left(\frac{\hat{f}_i - f_i}{f_i} \right)^2 .$$

We compare the test errors E_{test} on two types of stimulus-response data: smoothed and noisy. The prediction results using smoothed data are shown in Fig 2, with corresponding errors listed under the "Smoothed" category in Table E. Similarly, the errors for noisy data are presented in Fig B and under the "Noisy" category in Table E, respectively. The performance of both algorithms in predicting response data using noisy stimuli is similar to that using smooth stimuli, indicating that our algorithms are robust to noisy data with stochastic fluctuations, while noise levels can degrade model performance (see Table E). Although the input data (f_i, s_i) contains noise, the losses we design incorporate derivative approximations computed from smoothed data, ensuring that the neural network's output remains close to that obtained with smoothed inputs. Moreover, this approach guarantees stable long-term predictions of $f(t)$ without significant noise-induced fluctuations.

Table E. Test errors of NNs on Smoothed vs. Noisy stimulus-response data.

Models	Algorithm	Training set	E_{test} (%)
I	SIVM	Noisy	6.90%
		Smoothed	1.14%
	DIVM	Noisy	7.38%
		Smoothed	2.46%
II	SIVM	Noisy	3.45%
		Smoothed	1.36%
	DIVM	Noisy	1.27%
		Smoothed	0.54%
III	SIVM	Noisy	51.89%
		Smoothed	43.57%
	DIVM	Noisy	9.90%
		Smoothed	4.34%

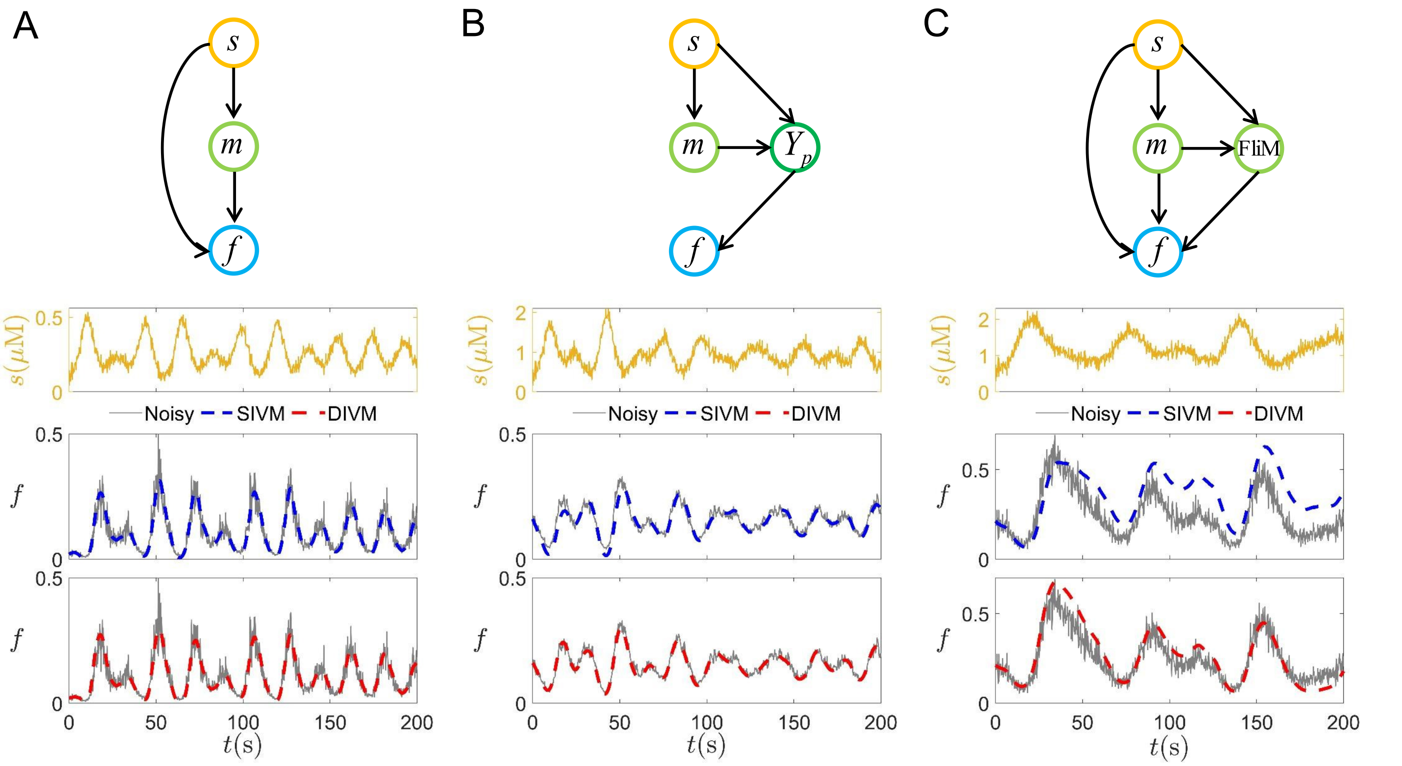

Fig B. Robustness test of SIVM and DIVM on *E. coli* models I-III using noisy ELCC signals. (A) Model I; (B) Model II; (C) Model III. Top row: the simplified topological relationships among external stimuli, internal variables, and tumbling fractions in the three different *E. coli* models. Second row: The external signal $s(t)$, obtained by adding Gaussian noise to ELCC stimuli with SNR following the uniform distribution $U(20, 22)$. The third row: the prediction of SIVM. The fourth row: the prediction of DIVM. Here, the response data (gray solid line) are the numerical solutions using noisy stimuli.

Appendix F. Extrapolation tests

In this section, we investigate the extrapolation capability of the algorithms, specifically the prediction accuracy when the value range and gradient range of test-set stimuli exceed those of the training set. Using Model II, we generate response data under given stimulus signals and construct noiseless training and test datasets. The training data is generated using PWC and LCC stimuli $s_0^{\text{train}}(t)$. The test datasets utilize some modifications of an ELCC stimulus $s_0^{\text{test}}(t)$, whose value range and gradient variation range remain consistent with the training data.

To get the algorithms' performance when either the value range or gradient range of the test data exceeds that of the training data, we modify $s_0(t)$ as follows:

- Value range extension: as shown in Fig C(A), we shift $s_0^{\text{test}}(t)$ upward by Δs such that:

$$s_\Delta(t) = s_0^{\text{test}}(t) + \Delta s.$$

  The corresponding response data are obtained via Model II. Each test set has a relative maximum deviation of the absolute stimulus value from the training set:

$$R_s = \frac{\max_{t\in[0,T]} |s_\Delta(t)| - \max_{t\in[0,T]} |s_0^{\text{train}}(t)|}{\max_{t\in[0,T]} |s_0^{\text{train}}(t)|}$$

- Gradient range extension: as shown in Fig D(A), $s_0(t)$ is compressed and the total duration time reduces from T to T_1 such that

$$s_T(t) = s_0^{\text{test}}(\frac{T}{T_1}t).$$

  The corresponding response data are using Model II. Each test set is associated with a relative maximum deviation of the absolute stimulus gradient from the training set:

$$R_{s'} = \frac{\max_{t\in[0,T_1]} |s'_T(t)| - \max_{t\in[0,T]} |s_0^{\prime,\text{train}}(t)|}{\max_{t\in[0,T]} |s_0^{\prime,\text{train}}(t)|}.$$

After training the neural networks using both SIVM and DIVM, we apply them to the two different categories of test sets described above. SIVM and DIVM test errors are correlated with the values of R_s and $R_{s'}$ of different data sets, as shown in Fig C(B) and Fig D(B). As the test data's deviation from the training range increases (both in value and the gradient of stimuli), the test errors of both algorithms grow. Regarding extrapolation capability, our framework maintains accurate predictions even when the gradient range expansion extends significantly beyond the training set. DIVM consistently outperforms SIVM in handling value range expansion. Notably, value range expansion exhibits stronger effects on the prediction accuracy of SIVM, while gradient range expansion exhibits stronger effects on the prediction accuracy of DIVM: as shown in Fig C(B) and Fig D(B), when $R_s = 0.49$ ($R_{s'} = 1.38$), SIVM's (DIVM's) error exceeds 10%.

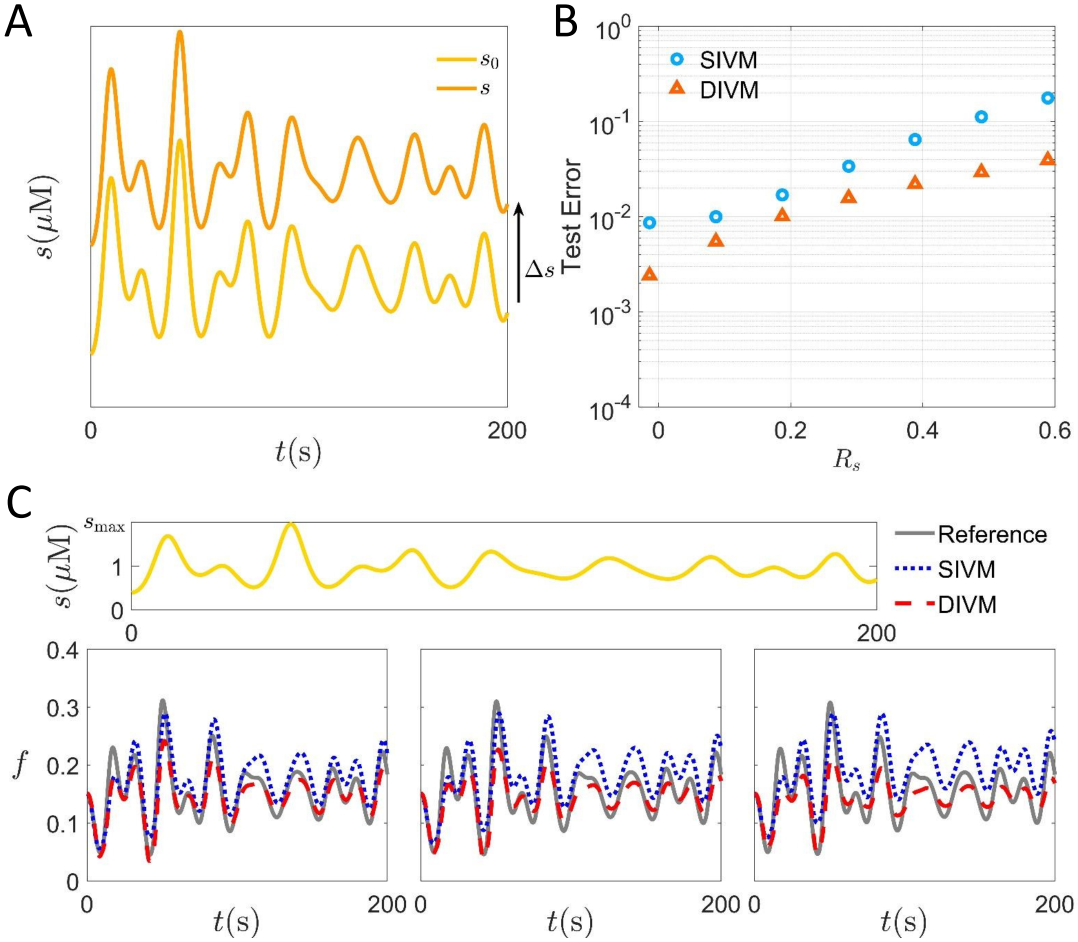

Fig C. Identify the feasible extrapolation range of s beyond the training ranges. External stimuli for extrapolation tests are shifted upward, as shown in (A). We gradually shift the stimulus signal upward by Δs, where Δs takes values of $\Delta s = \{0, 0.2, 0.4, 0.6, 0.8, 1, 1.2\}$ μM. The relative maximum deviation of the absolute stimulus value R_s is correlated with SIVM and DIVM test errors in (B). The subplots in (C) from left to right correspond to the three error points for $R_s = 0.39$, $R_s = 0.49$, and $R_s = 0.59$ in (B).

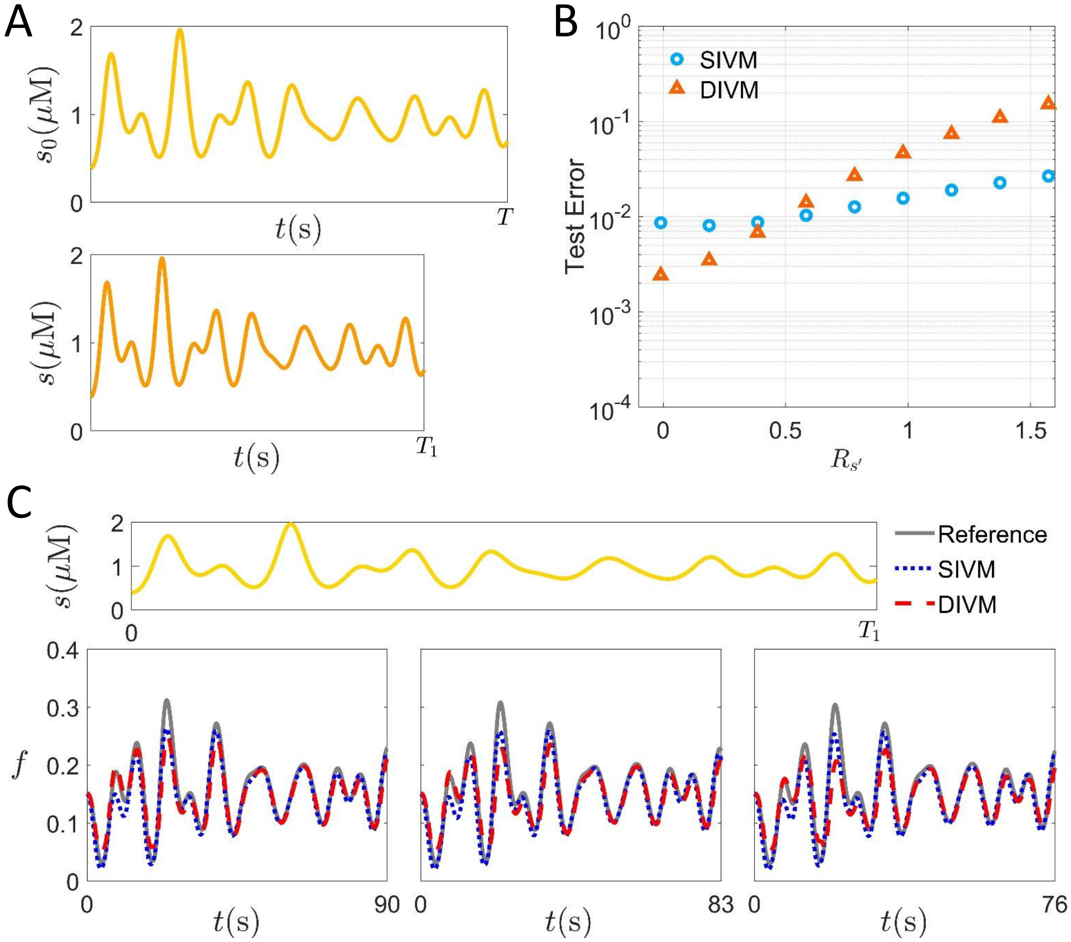

Fig D. Identify the feasible extrapolation range of s' beyond the training ranges. External stimuli for extrapolation tests are compressed in time, as shown in (A). We gradually compress the $T = 200$ s stimulus signal to $T_1 = 100$ s, making the signal's relative maximum deviation of the absolute stimulus gradient $R_{s'}$ increase to 1.57. $R_{s'}$ is correlated with SIVM and DIVM test errors in (B). The error points from left to right correspond to $T_1 = \{200, 167, 143, 125, 111, 100, 90, 83, 76\}$ s respectively. The subplots in (C) from left to right correspond to the three error points for $R_{s'} = 1.18$, $R_{s'} = 1.38$, and $R_{s'} = 1.57$ in (B).

Supplementary Figures

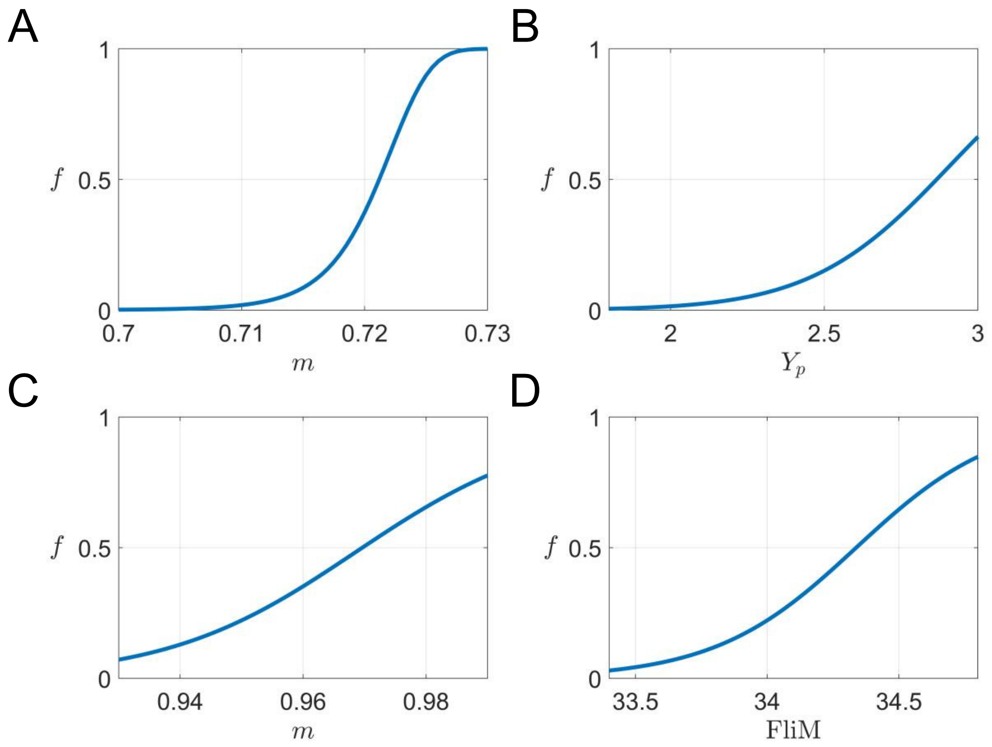

Fig E. Response f of Model I-III depends monotonically on internal-state variables. (A) shows the curve of f varying with m in Model I, with fixed $s = 0.25$. (B) shows the curve of f varying with Y_p in Model II. (C) shows the curve of f varying with m in Model III, with fixed $s = 0.5$ and FliM $= 34$. (D) shows the curve of f varying with FliM in Model III, with fixed $s = 0.5$ and $m = 0.95$.

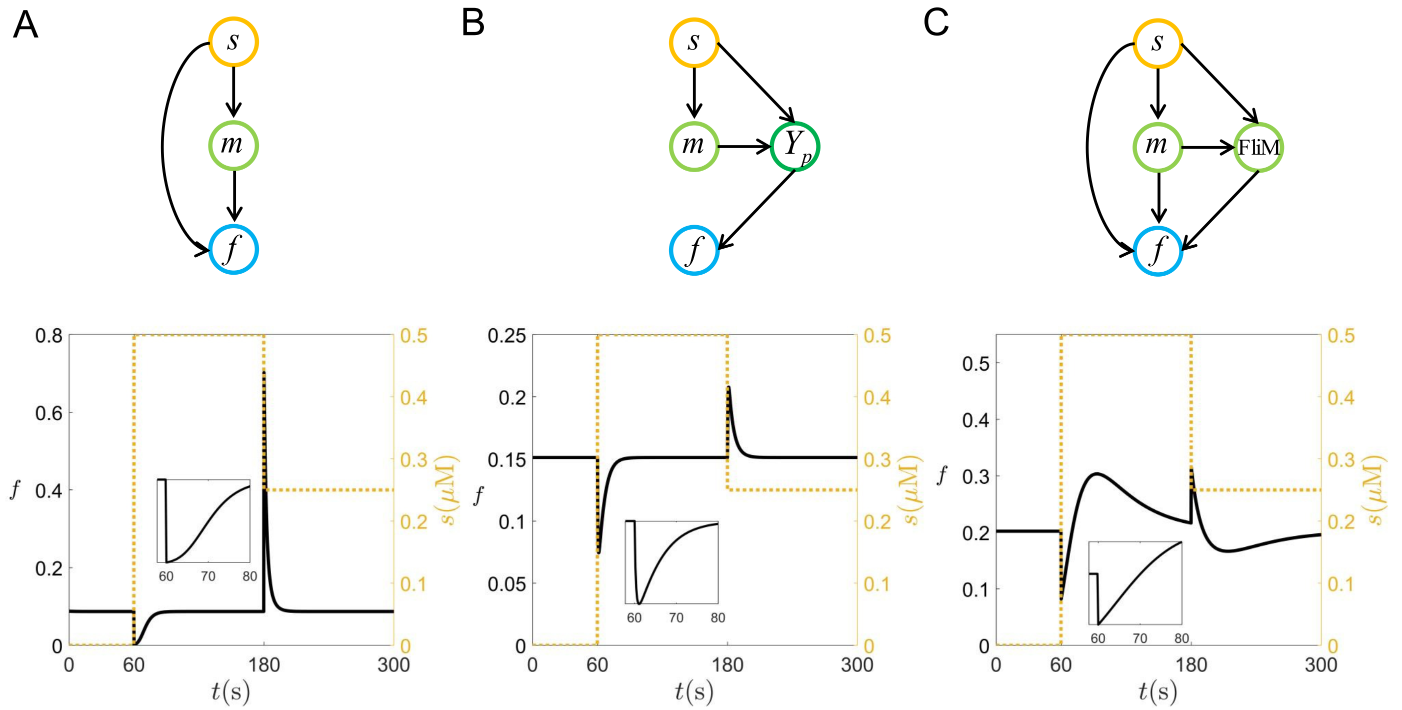

Fig F. The response f of Model I-III to a sudden change $s(t)$. (A-C), top line, illustrates the simplified topological relationships among external stimuli, internal variables, and tumbling responses in the *E. coli* model. $x \to y$ denotes that x has an influence on y, which can encompass both promotion and inhibition. The bottom line demonstrates the tumbling fraction (as indicated in the black line) under a step signal (represented by the yellow dotted line).

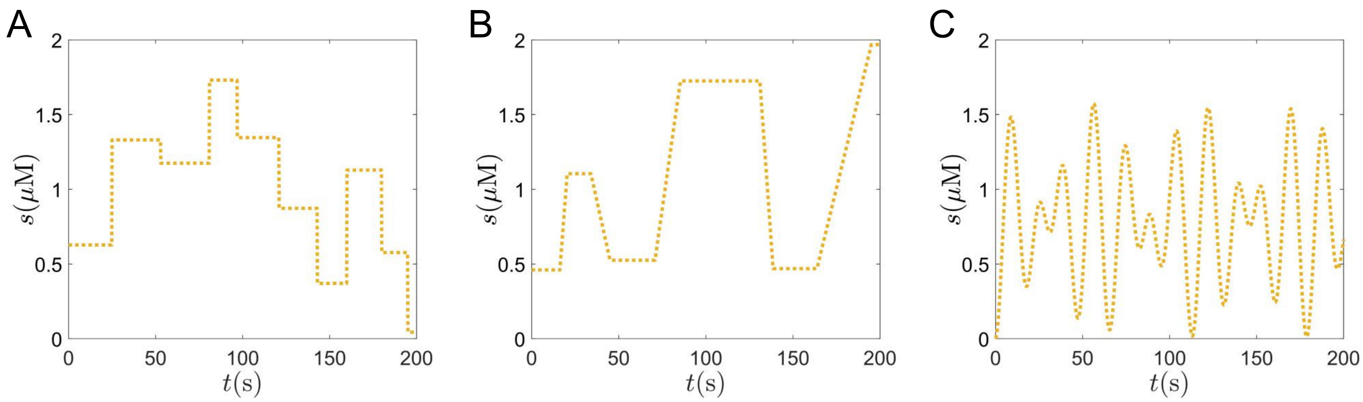

Fig G. The three types of stimuli that form the training set. (A-C) illustrate piecewise constant (PWC), piecewise constant and linear (PWCL), and linear combination of cosine (LCC) signals, respectively.

References

1. Xue C. Macroscopic Equations for Bacterial Chemotaxis: Integration of Detailed Biochemistry of Cell Signaling. Journal of Mathematical Biology. 2015;70(1-2):1–44. doi:10.1007/s00285-013-0748-5.

2. Si G, Wu T, Ouyang Q, Tu Y. Pathway-Based Mean-Field Model for *Escherichia Coli* Chemotaxis. Physical Review Letters. 2012;109(4):048101. doi:10.1103/PhysRevLett.109.048101.

3. Xue X, Xue C, Tang M. The Role of Intracellular Signaling in the Stripe Formation in Engineered Escherichia Coli Populations. PLOS Computational Biology. 2018;14(6):e1006178. doi:10.1371/journal.pcbi.1006178.

4. Tu Y, Shimizu TS, Berg HC. Modeling the Chemotactic Response of *Escherichia Coli* to Time-Varying Stimuli. Proceedings of the National Academy of Sciences. 2008;105(39):14855–14860. doi:10.1073/pnas.0807569105.

5. Min TL, Mears PJ, Golding I, Chemla YR. Chemotactic Adaptation Kinetics of Individual Escherichia Coli Cells. Proc Natl Acad Sci USA. 2012;109(25):9869–74. doi:10.1073/pnas.1120218109.

6. Giometto A, Altermatt F, Maritan A, Stocker R, Rinaldo A. Generalized Receptor Law Governs Phototaxis in the Phytoplankton Euglena Gracilis. Proc Natl Acad Sci USA. 2015;112(22):7045–50. doi:10.1073/pnas.1422922112.

7. Rossi M, Cicconofri G, Beran A, Noselli G, DeSimone A. Kinematics of Flagellar Swimming in Euglena Gracilis: Helical Trajectories and Flagellar Shapes. Proc Natl Acad Sci USA. 2017;114(50):13085–13090. doi:10.1073/pnas.1708064114.

8. Giuliani N, Rossi M, Noselli G, DeSimone A. How Euglena Gracilis Swims: Flow Field Reconstruction and Analysis. Phys Rev E. 2021;103(2-1):023102. doi:10.1103/PhysRevE.103.023102.

9. Masson JB, Voisinne G, Wong-Ng J, Celani A, Vergassola M. Noninvasive Inference of the Molecular Chemotactic Response Using Bacterial Trajectories. Proc Natl Acad Sci USA. 2012;109(5):1802–7. doi:10.1073/pnas.1116772109.
