# Supplementary material for: Identification of the governing equation of stimulus-response data for run-and-tumble dynamics
Source: PLoS Comput Biol. 2025 Aug 5;21(8):e1013287. doi: 10.1371/journal.pcbi.1013287 (PMC12338844; doi:10.1371/journal.pcbi.1013287)
Supplement: Attachment — Submitted filename: Response to Reviewers.pdf [file pcbi.1013287.s003.pdf]

Reply to the Reviewers

Re: Manuscript ID PCOMPBIOL-D-25-00309
"Identification of the governing equation for run-and-tumble dynamics from stimulus-response data"
Shicong Lei, Yu'an Li, Zheng Ma, H. P. Zhang, Min Tang
PLOS Computational Biology

Dear Prof. Calina Copos and Prof. Feilim Mac Gabhann,

Thank you for your letter containing two reviewer reports on our manuscript PCOMPBIOL-D-25-00309.

Both Reviewer #1 and Reviewer #2 recommended the manuscript for publication in *PLOS Computational Biology*, while providing valuable suggestions for further enhancing its quality.

We wish to express our sincere gratitude to both Reviewers for their insightful comments. We have carefully addressed all questions raised by the reviewers and revised the manuscript accordingly, as detailed below. By incorporating their suggestions, we believe we have significantly strengthened the quality of our manuscript.

Sincerely,
Min Tang

List of main changes

1. We have rewritten the "Author Summary" to present the significance or possible implications of our work simply and objectively, making it accessible to a wide audience that includes both scientists and non-scientists.
2. We have restructured the "Introduction" section and clarified that our framework is developed based on general stimulus-response dynamics without relying on organism-specific assumptions. The models for *E. coli* chemotaxis and *Euglena gracilis* photo-response are then treated as two parallel applications of this general framework. The detailed discussion of the *E. coli* models has been moved to Line 146-171 on page 6 in the "Methods" section. The review on stimulus types employed in previous experimental studies has been moved to Line 510-516 on page 18 in the "Results" section.
3. We have added a *Remark* in Line 135-144 on page 6 to explain the monotonicity assumption of response functions (F) on internal variables.
4. We have restructured the "Methods" section to emphasize that our method is applied in parallel to two different microorganisms' examples. We have rewritten Subsection 3: Photo-response movement in algae, adopting a similar narrative structure for both microorganisms' examples. We summarized the current understanding of their internal phototaxi/chemotaxi reaction pathways and the corresponding behavioral responses.
5. We have expanded the discussion on the implications of the SIVM and DIVM performance. The threshold of the gradient of the signal can indicate some parameter values in the internal dynamics of *Euglena gracilis* phototaxis. Please see Line 423-430 on page 16.
6. We have comprehensively restructured the "Discussion" section. The arrangement is to first summarize the algorithm, then show algorithmic strengths and innovations, and finally discuss the potential future research directions.
7. We have added a brief derivation of the theoretical threshold in Line 447-461 on page 16 and compared the theoretical results with the numerical results in Line 478-479 on page 18. We also added a new appendix (Appendix C) to the Supporting Materials with all details of the derivation.
8. We have added the discussion on the robustness of our framework under complex stimuli (Line 374-385 on page 13-14) and the extrapolation performance beyond the training ranges (Line 491-508 on page 18). More details are in the new sections (Appendix E and F) of the Supporting Materials.
9. We have redesigned Fig 4. The revised subplot A clarifies the stimulus signal compression process, while the revised subplot B incorporates the test error of both algorithms.

Reply to Reviewer #1

We extend our gratitude to the Reviewer for the positive evaluation of our work and sincerely appreciate the insightful comments and suggestions, to which we reply below.

Reviewer #1, comment #1:

The study builds on the well-known dynamics of E. coli chemotaxis, with Model I focusing on the slow dynamics of m, Model II incorporating the slow dynamics of m and the fast dynamics of Y_p, and Model III including the slow dynamics of both m and FliM. Given the differences in timescale separation among the intermediate variables in the three models, the performance of SIVM and DIVM on the generated data is not surprising.

The potentially novel insight is that this method could possibly extract information about the internal dynamics of Euglena gracilis phototaxis. For example, the varying performance of SIVM and DIVM at different $s'_{\max}$ values may suggest that the phototaxis pathway can also be described by two variables—one fast and one slow. The authors should expand on this point and connect it to what is known about the phototaxis pathway in Euglena gracilis.

Response: #1.1

We appreciate the Reviewer's insightful comments regarding the potential novel interpretation of our results. Following the suggestion, we have expanded the discussion on the implications of the SIVM and DIVM performance for understanding the internal dynamics of *Euglena gracilis* phototaxis. In particular, we emphasize how the different behaviors of SIVM and DIVM across various $s'_{\max}$ values indicate the possible existence of two key internal variables evolving with distinct timescales. We have also added a brief discussion connecting this finding to the current biological knowledge of *Euglena* photo reception and signal transduction mechanisms.

Please see Line 423-430 on page 16 where we expand the discussion on the implications.

Reviewer #1, comment #2:

The assumption of monotonic dependence of response functions (F) on internal variables (m, n) is critical for inverting the system. While this assumption holds for the well-studied E. coli chemotaxis pathway, no validation is provided for experimental systems like E. gracilis phototaxis. The authors should include sensitivity analyses or empirical evidence to justify this assumption. If monotonicity cannot be verified, alternative approaches should be discussed.

Response: #1.2

We appreciate the Reviewer for emphasizing this crucial point. Indeed, the monotonicity of the response function F with respect to internal variables is essential for inverting the function. While this assumption is valid for *E. coli* chemotaxis, its applicability to *Euglena gracilis* phototaxis remains unclear. The core concept of our reformulation is as follows: the quantities m and n are unknown; they potentially represent the concentration of a specific protein or a function thereof. This approach allows m and n to be defined flexibly, such that they do not necessarily represent the concentration of a specific protein. We assume the protein concentration is c, and define $m = \phi(c)$. By making no assumptions about the specific form of ϕ, we can always posit the existence of a function ϕ such that F is monotonic with respect to $\phi(c)$, even if F is non-monotonic with respect to c itself.

Please see *Remark* in Line 135-144 on page 6.

Reviewer #1, comment #3:

The training data rely on synthetic stimuli (PWC, PWCL, LCC). However, real-world signals may exhibit more complex patterns (e.g., stochastic fluctuations). The paper should discuss the framework's robustness to non-synthetic stimuli and whether extrapolation beyond the training ranges (e.g., higher $s'_{\max}$) is feasible.

Response: #1.3

We thank the Reviewer for raising this important point regarding the robustness of our framework under complex stimuli and the extrapolation performance beyond the training ranges. Since in LCC, the period and amplitude are chosen randomly, it can represent general smooth signals. In Line 374-385 on page 13-14, we

examine the framework's performance under noisy and stochastic stimuli. Our results show that the method remains robust when trained and tested on stimuli with moderate stochastic fluctuations, although extreme noise levels can degrade model performance. In Line 491-508 on page 18, we discuss the extrapolation ability of the trained models for larger $s_{\max}$ and $s'_{\max}$ values beyond the training range. Our algorithms maintain accurate predictions even when the gradient range expansion extends significantly beyond the training set. DIVM consistently outperforms SIVM in handling value range expansion.

More details are in the new sections (Appendix E and F) of the Supporting Materials.

Reviewer #1, comment #4:

A critical value of the stimulus signal gradient, 0.5 µM/s, *is identified, beyond which SIVM becomes inapplicable. However, this specific value lacks meaningful context without further discussion. The authors should relate this value to the parameter values used in the chemotaxis model to provide deeper insight.*

Response: #1.4

We thank the Reviewer for pointing out the need for a deeper understanding of the identified critical stimulus gradient value (0.5 µM/s). We have added a new section (Appendix C) in the Supporting Materials, where we analytically derive the relationship between the critical gradient and key parameters of the chemotaxis model, such as adaptation rates and receptor sensitivity. Furthermore, we compare the theoretical threshold with the numerical results, confirming their consistency. This additional analysis highlights the biological relevance of the critical value and clarifies the conditions under which the SIVM is valid.

Please see the brief derivation of the theoretical threshold in Line 447-461 on page 16, and the numerical comparison is in Line 478-479 on page 18. We also added a new appendix (Appendix C) in the Supporting Materials, where details of the theoretical derivation are shown.

Reviewer #1, comment #5:

Citations for the parameter values in the supplemental tables should be provided.

Response: #1.5

We thank the Reviewer for bringing this to our attention. We have added citations for the parameter values in Tables S1-S4 of Appendix A in the Supporting Materials.

Reviewer #1, comment #6:

Minor comment

Line 241: Define "PWC" upon its first occurrence.

Response: #1.6

We thank the Reviewer for noting this point. We define the "PWC" in Line 320 on page 10 upon its first occurrence.

Reply to Reviewer #2

We thank the Reviewer for the precise summary of our work and the positive words. Below, we will address the comments in detail.

Reviewer #2, comment #1:

The two contexts to which the model is applied are placed on different levels in different sections of the manuscript. Sometimes it seems that the model was determined by studying Escherichia Coli and then applied to Euglena, sometimes instead the message is conveyed that the model was determined on more general dynamics, relating to general microorganisms and then applied to the two contexts in a parallel way, as if they were two examples at the same level. I believe that this aspect should be clarified by choosing one of the two approaches and following it with the structure of the manuscript, with consecutive or parallel sections depending on the approach chosen.

Response: #2.1

We thank the Reviewer for pointing out the unclearance of the structure when presenting the two microorganism examples. We have revised the manuscript to clarify that our framework is developed based on general stimulus-response dynamics without organism-specific assumptions. The models for *E. coli* chemotaxis and *Euglena gracilis* photo-response are treated as two parallel applications of this general framework. This clarification has been explicitly stated in the "Introduction" section, and the manuscript structure has been adjusted to maintain consistency throughout.

We restructured the "Introduction" section as follows: beginning with biological phenomena of external stimuli response and establish a generalized modeling framework (Paragraphs 1-2), then highlighting the challenges in solving for H and F (Paragraphs 3-4) and presenting our proposed algorithm (Paragraph 5), afterwardemonstrating its applications in *Escherichia Coli* and *Euglena* systems respectively (Paragraphs 6-7), and finally concluding with comparative advantages of our method over other neural network approaches (Paragraphs 8).

Reviewer #2, comment #2:

In the "Introduction", we focus a lot on Escherichia Coli. In the behavioral response models are cited that take into consideration different forms $f = F(m_1, s)$, $f = F(m_1)$, and $f = F(m_1, m_2, s)$ which, unlike the general model with an unspecified number J of m, considers a very precise number that depends on what those m represent in the case of Escherichia Coli. These factors are not mentioned here, leading to a question about the necessity of entering the specific case of Escherichia Coli at this point in the discussion. Note that these factors are then specified in the Methods subsection "E. coli chemotaxis and related models". In my opinion, this creates a lot of expository confusion. I find the authors need to ask themselves what information they want to include in the "Introduction" and what in the "Methods", and clearly differentiate the sections in this sense.

Response: #2.2

We appreciate the Reviewer's suggestion to better differentiate the content of the "Introduction" and "Methods" sections. Following the suggestions of both Reviewers, we have rewritten the "Introduction" and focused on the general modeling strategy, without specify the organism. The detailed discussion of the *E. coli* models, including specific forms of F and internal variables, has been moved to the "Methods" section. This restructuring improves the clarity of the manuscript and ensures a clear separation of the general theoretical background and organism-specific details.

Reviewer #2, comment #3:

In the "Methods" section, the structure is as follows:
Section: Methods

- *Subsection 1: E. coli chemotaxis and related models*
- *Subsection 2: Experiments of Euglena gracilis*
- *Subsection 3: The modeling framework*
- *Subsection 4: Implementation*

The modeling framework is related to both, it is not clear to me the choice to first present the models relating to Escherichia Coli, then move on to the experiments on Euglena, then return to the modeling framework... I find that the logical flow is thus interrupted. In my opinion, it would be more relevant to open with a general model, enter into the specifics introduced once focused on Escherichia Coli, then move on to explain how this structure fits into the context of Euglena, and end with the implementation. I suggest improving the structure of this section.

Response: #2.3

We have rearranged the section "Methods" and the new structure is as follows:

- Subsection 1: The modeling framework
- Subsection 2: Chemotaxis models in *E. coli*
- Subsection 3: Photo-response movement in algae
- Subsection 4: Implementation

To emphasize that our method is applied in parallel to these two microorganisms' examples, we rewrote Subsection 2 and 3 and adopted a similar narrative structure at the beginning of each subsection.

Reviewer #2, comment #4:

In my opinion, it is not of particular interest to put the questions expressed in the "Author summary" in a separate section. If the introduction guides the reader through the formulation of the model and its foundations, I think this would benefit from being enriched by the content of the "Author summary".

Response: #2.4

According to the journal's requirements for the "Author Summary": "This text is subject to editorial change, should be written in the first-person voice, and should be distinct from the scientific abstract. Aim to highlight where your work fits within a broader context; present the significance or possible implications of your work simply and objectively; and avoid the use of acronyms and complex terminology wherever possible. The goal is to make your findings accessible to a wide audience that includes both scientists and non-scientists." We have rewritten the Author Summary accordingly. I hope it better meets the requirements.

Reviewer #2, comment #5:

In my opinion, the "Discussion" is too brief. It should contain a summary of the procedures followed and the model presented. It is necessary to clearly and schematically highlight the novelties and strengths of the work, underlining the results obtained. Finally, the "Discussion" should clarify the next steps and open questions. I suggest significantly expanding this section, since the contents to be included are already presented throughout the manuscript, but require a general closing and summary.

Response: #2.5

We have expanded the "Discussion" section by first summarize the algorithm summary, highlight algorithmic strengths and innovations, and finally disucss potential future research directions. Please see the new discussion section.

Reviewer #2, comment #6:

Minor observations

A) The abstract is well written and clarifies the theme of the manuscript well. As my only observation, I would suggest improving the clarity of the passage "... without reconstructing the ODE system that models the intracellular chemical reactions. The structure of the model takes into account the general structure of ODEs that model the intracellular biochemical reactions and ..."

For me, the repetition confuses a little the difference that the authors want to highlight here. I would express it in a more concise way.

E.g. "We introduce a neural network-based model that identifies the governing equations of run-and-tumble dynamics. The model accounts for the nonlinear functions that describe movement responses to intracellular

biochemical reactions, incorporating the general structure of ODEs that represent these reactions without explicitly reconstructing them.)

B)Please check the English. There are also some typos or word repetition throughout the manuscript.

Response: #2.6

We appreciate the Reviewer for the helpful suggestion to enhance the expression in the "Abstract". We have modified accordingly, carefully proofread the manuscript and corrected some spelling errors. We hope the current version is satisfactory.
