# Supplementary material for: Identification of the governing equation of stimulus-response data for run-and-tumble dynamics
Source: PLoS Comput Biol. 2025 Aug 5;21(8):e1013287. doi: 10.1371/journal.pcbi.1013287 (PMC12338844; doi:10.1371/journal.pcbi.1013287)
Supplement: Attachment — Submitted filename: review_D-25-00309.odt [file pcbi.1013287.s002.odt]

The manuscript falls within the macro-area of neural network-based models for determining movement mechanisms in biology. In particular, the model presented here aims to identify governing equations of microorganisms, accounting for the ODE system that models the intracellular chemical reactions that lead to the run and tumble dynamics, but without reconstructing it. The Authors show two different applications, one to Escherichia Coli and the other to Euglena gracilis cells, testing on experimental data and showing the efficiency of the model presented.
Although my area of expertise is more closely related to the application field of the manuscript and the mathematical models used as the structural basis for the neural network-based models presented, I find the adopted methodology to be coherent and relevant. The results presented are innovative, significant, and aligned with the characteristics of this journal.
Despite the high quality of the contents of the work and the general exposition clarity of the individual sections, I find that some exposition choices need to be reviewed, as in my opinion the logical thread of the discussion is sometimes broken, making the fluidity that is instead found in the determination of the model difficult to access.
I therefore recommend a minor revision.
## Major Issues
The most important thing to fix in my opinion is the general structure of the discussion.
I highlight the (in my opinion) major critical issues below.
The two contexts to which the model is applied are placed on different levels in different sections of the manuscript. Sometimes it seems that the model was determined by studying Escherichia Coli and then applied to Euglena, sometimes instead the message is conveyed that the model was determined on more general dynamics, relating to general microorganism,s and then applied to the two contexts in a parallel way, as if they were two examples at the same level. I believe that this aspect should be clarified by choosing one of the two approaches and following it with the structure of the manuscript, with respectively consecutive or parallel sections depending on the approach chosen.
In the “Introduction” we focus a lot on Escherichia Coli. In the behavioral response models are cited that take into consideration different forms f = F (m1, s), f = F (m1), f = F (m1, m2, s) which, unlike the general model with an unspecified number J of m, considers a very precise number that depends on what those m represent in the case of Escherichia Coli. These factors are not mentioned here, leading to a question about the necessity of entering the specific case of Escherichia Coli at this point of the discussion. Note that these factors are then specified in the Methods subsection “E. coli chemotaxis and related models”. In my opinion this creates a lot of expository confusion. I find the authors need to ask themselves what information they want to include in the “Introduction” and what in the “Methods” and clearly differentiate the sections in this sense.
In the “Methods” section the structure is the following.
Section: Methods
subsection 1: E. coli chemotaxis and related models
subsection 2:  Experiments of Euglena gracilis
subsection 3:	The modeling framework
subsection 4:	Implementation
The modeling framework is related to both, it is not clear to me the choice to first present the models relating to Escherichia Coli, then move on to the experiments on Euglena, then return to the modeling framework... I find that the logical flow is thus interrupted. In my opinion it would be more relevant to open with a general model, enter into the specifics introduced once focused on Escherichia Coli, then move on to explain how this structure fits into the context of Euglena and end with the implementation. I suggest to improve the structure of this section.

In my opinion it is not of particular interest to put the questions expressed in the “Author summary” in a separate section. If the introduction guides the reader through the formulation of the model and its foundations, I think this would benefit from being enriched by the content of the “Author summary”.
In my opinion the “Discussion” is too brief. It should contain a summary of the procedures followed and the model presented. It is necessary to clearly and schematically highlight novelties and strengths of the work, underlining the results obtained. Finally the “Discussion” should clarify the next steps and open questions. I suggest significantly expanding this section, since the contents to be included are already presented throughout the manuscript, but require a general closing and summary.
## Minor observations
The abstract is well written and clarifies the theme of the manuscript well. As my only observation, I would suggest improving the clarity of the passage “… without reconstructing the ODE system that models the intracellular chemical reactions. The structure of the model takes into account the general structure of ODEs that model the intracellular biochemical reactions and …”
For me, the repetition confuses a little the difference that the authors want to highlight here. I would express it in a more concise way.
E.g. “We introduce a neural network-based model that identifies the governing equations of run-and-tumble dynamics. The model accounts for the nonlinear functions that describe movement responses to intracellular biochemical reactions, incorporating the general structure of ODEs that represent these reactions without explicitly reconstructing them.)
Please check the English, There are also some typos or word repetition throughout the manuscript.

In congratulating the authors for the good results obtained, I hope these observations help them to bring the exposition to the level of the contents.
##
